# Prevalence and Predictors of Anxiety and Depression Symptoms during the COVID-19 Pandemic and Compliance with Precautionary Measures: Age and Sex Matter

**DOI:** 10.3390/ijerph17144924

**Published:** 2020-07-08

**Authors:** Ioulia Solomou, Fofi Constantinidou

**Affiliations:** 1 Center for Applied Neuroscience, University of Cyprus, Nicosia 1678, Cyprus; isolom01@ucy.ac.cy; 2 Department of Psychology, University of Cyprus, Nicosia 2109, Cyprus

**Keywords:** COVID-19, psychological effects, anxiety, depression, precautionary measures, quality of life

## Abstract

Effective management of the global pandemic caused by Severe Acute Respiratory Syndrome Coronavirus 2 (also known as COVID-19), resulted in the implementation of severe restrictions in movement and enforcement of social distancing measures. This study aimed to understand and characterize the psychosocial effects of the COVID-19 pandemic in the general population and to identify risks and protective factors that predict changes in mental health status. In addition, the study investigated compliance with precautionary measures (PM) to halt the spread of the virus. The online anonymous survey collected information on sociodemographic data, compliance with PM, quality of life (QOL), and mental health via the Generalized Anxiety Disorder-7 (GAD-7) and Patient Health Questionnaire-9 (PHQ-9). A total of 1642 adult participants (71.6% women, 28.4% men) completed the survey in the European island country, Cyprus. A large percentage (48%) reported significant financial concerns and 66.7% significant changes in their QOL. About 41% reported symptoms associated with mild anxiety; 23.1% reported moderate-severe anxiety symptoms. Concerning depression, 48% reported mild and 9.2% moderate-severe depression symptoms. Women, younger age (18–29), student status, unemployment status, prior psychiatric history, and those reporting greater negative impact on their QOL, were at higher risk for increased anxiety and depression symptoms (*p* < 0.05). The youngest age group and males also reported lower levels of compliance with PM. Higher compliance with PM predicted lower depression scores (*p* < 0.001) but higher anxiety for measures related to personal hygiene. The results of this study provide important data on the effects of the COVID-19 outbreak on mental health and QOL and identify a variety of personal and social determinants that serve as risks and protective factors. Furthermore, it has implications for policy makers demonstrating the need for effective mental health programs and guidance for the implementation of PM as a public health strategy.

## 1. Introduction

On January 30, the World Health Organization (WHO) declared the severe acute respiratory syndrome coronavirus 2 (SARS-CoV-2, also known as COVID-19) outbreak a public health emergency of international concern. In only one month, at least one thousand cases had been reported in five different countries [1]. On March 11, WHO declared the disease caused by COVID-19 a pandemic due to its widespread and rapid rate of transmission. This is the third respiratory pandemic illness of the 21st century. The first was the severe acute respiratory syndrome (SARS) in 2003, which resulted in more severe symptoms and a higher death rate than COVID-19. However, the rapidly forming situation of the COVID-19, the rate of transmission, and the number of people infected in over 150 countries, to date, created an unprecedented situation of global proportions [2,3]. 

Health pandemics mobilize all resources of local and national health care systems in an effort to manage those infected and prevent the spread of the disease. Few health care systems acknowledge the significance of mental health intervention as a key pillar in effective disease management [4]. The purpose of the present study was to contribute to the very limited research on the psychosocial effects of the COVID-19 pandemic in the general population. The study took place primarily in Cyprus, an island country in the Eastern Mediterranean region and member of the EU. With a population of about 1,205,489 people [5], Cyprus boasts a very high literacy level, and, like other southern Mediterranean nations, it is characterized by strong social and family networks [6]. 

In light of lack of vaccination or community immunization and in order to limit the rate of the spread of the infection (ρ0) during the escalation phase of the pandemic, several governments have imposed movement restrictions and social distancing measures. The government of Cyprus has received praise on the effective management of the crisis [7]. The government moved swiftly by immediately closing down schools and a gradual closing of businesses. Within a week of the first case on March 9, airports and all access to the country were closed, and within two weeks severe lock down and social distancing measures were enforced. In a review article that included 24 studies, Brooks and associates (2020) caution about the potential psychological impact of such measures. While they may be effective in managing the spread of a pandemic, Brooks et al. (2020) assert that their duration should be limited and only for the absolute period necessary to control the climax of the pandemic [8].

The 2002 SARS pandemic generated a rich bibliography on the psychological and psychosocial implications for different high-risk groups, such as frontline healthcare workers, SARS patients, or even quarantined individuals who stayed home to avoid cross-infection [9,10,11,12]. Specific results from the mentioned studies and from a recently published study on health care workers treating patients with COVID-19 indicate that frontline workers are likely to experience higher levels of fear of contagion, stress, and psychological distress [10,13,14].

While those working in the front lines and those infected with the virus may be at a high risk for lingering psychological problems, research indicates that the non-infected general population during the SARS epidemic showed substantial psychiatric morbidities. One explanation was that younger people experienced a greater degree of self-blame [15]. 

There are only a couple of published to date on the effects of the COVID-19 pandemic on the general public. Wang and associates [16] conducted a survey in China to address the psychological problems during the initial stage of the COVID-19 outbreak, using the Depression, Anxiety and Stress Scale (DASS-21). Out of the 1211 participants, 53.8% rated the psychological impact of the outbreak as moderate or severe; 28.8% reported moderate to severe anxiety symptoms; 16.5% reported moderate to severe depressive symptoms; and 8.1% reported moderate to severe stress levels [16]. The authors concluded that COVID-19 may result in greater psychological impact than SARS; however, more research is required to characterize the effects of the current pandemic to the general public.

Another study from China focused on university students. The study focused only on anxiety symptoms. Of the 7143 participants, 21.3% reported mild anxiety, 2.7% moderate anxiety, and 0.9% severe anxiety [17]. Family income stability, living with parents and overall social support were protective factors against anxiety. 

The current study contributes to the growing body of evidence on the psychological impact of the current COVID-19 pandemic in the general population during the escalation of the pandemic. This is the first study conducted in the Mediterranean region and from a fairly homogeneous sample. Specifically, the study aimed to determine the prevalence and degree of self-reported psychological symptoms and identify demographic, environmental, and individual protective and risk factors. In addition, the study investigated compliance to recommended precautionary measures to prevent the spread of the disease. We hypothesized that government-imposed lock-down measures, along with the current effects of the global pandemic, will have a significant effect on quality of life and psychological well-being of the Cypriot population. Furthermore, we anticipated that factors, such as age and sex, as well as pre-existing psychiatric history, living conditions, and financial concerns, would be significant predictors of psychological and mental well-being.

## 2. Materials and Methods

### 2.1. Sample and Setting 

The study was approved by the Cyprus Bioethics Committee ΕΕΒΚ ΕΠ 2020.01.54. We used a mixed sampling random procedure and a snowball sampling procedure to recruit a respectful sample size and the results could be generalized to the rest of the population. The only inclusion criterion was that the participant had to be 18 years of age or older. Concerning the recruitment methods, we used the basic Facebook approach, posting messages on multiple targeted Facebook groups with users over 18 years old, as well as an anonymous email approach. Specifically, an email with the survey information and the link for participation was sent to entire University of Cyprus community (students, faculty, and staff), and they were encouraged to pass it on to their networks.

### 2.2. Data Collection Procedure

The survey was conducted online to maximize reach and ensure anonymity. Participants had the option to complete the questionnaires in Greek or English through the Research Electronic Data Capture (REDCap) online survey platform [18]. Information about the study objectives and the procedure was given to the participants followed by their consent to participate. Volunteers were informed that their participation was anonymous and voluntary and that they could, at any point, close the window and discontinue participation; none of the incomplete data would be used in the analyses. Data were collected over 1 week from April 3 to April 9, two weeks after the strict lock down measures and movement restrictions were implemented. 

### 2.3. Survey Development and Measures 

The online survey was developed in order to be brief (under 10 min) and at the same time obtain important information relevant to the objectives of the study. Since participation was voluntary, and the data obtained anonymous and untraceable, it was assumed that participants responded with honesty and without fear of judgment. The 56 questions were presented in sections to maximize organization and minimize fatigue. The text was set in black Arial font and participants could adjust the font size according to their preference using the button at the top right of each survey page. Information about the page number and the total number of pages was displayed on the screen. The survey was also accessible via mobile devices. 

The questionnaire was comprised of four major sections: sociodemographic, COVID-19 knowledge and compliance with precautionary measures (PM), quality of life (QOL), and mental health questions. First, we conducted a pilot study with 25 volunteers to assess the internal consistency of the questionnaires. Reliability index (Cronbach’s α coefficient) for Compliance with PM against COVID-19 for Generalized Anxiety Disorder-7 and Patient Health Questionnaire-9 was 0.79, 0.91 and 0.88, respectively, demonstrating good internal consistency.

#### 2.3.1. Sociodemographic Data

Sociodemographic data included information about sex, age, current country of residence, country of permanent residence, current employment, and/or student status. Additional sociodemographic questions relevant to the COVID-19 situation included: physical/social distancing measures taken at their workplace, type of current employment (i.e., if they are frontline workers) and whether the individual or a first degree relative was trapped broad. Medical history information questions included prior anxiety or depression history, recent (within 2 weeks) communication with their medical doctor (and reason for communication), and whether they had contracted COVID-19 or came in contact with a confirmed case.

#### 2.3.2. Compliance with Precautionary Measures (PM) against COVID-19

To assess compliance with PM against COVID-19, we developed 12 questions measuring key aspects of the prevention guidelines implemented by the WHO, the CDC, the ECDC, and the Republic of Cyprus. Examples include, hand washing, avoiding hand contact, physical distancing, etc. Respondents rated the frequency of implementation of these measures in the two weeks prior to their participation using a 5-point Likert scale rating from Never to Always Cronbach’s α for the entire sample was 0.76, indicating good internal consistency. 

#### 2.3.3. Quality of Life (QOL)

To assess the QOL, we included 5 independent questions measuring the key aspects QOL. Examples include finances, personal health, and satisfaction with life. Respondents were asked to rate on a 5-point scale how dissatisfied or satisfied they were with their QOL in the past 14 days.

#### 2.3.4. Mental Health Status

Mental health status was measured using the General Anxiety Disorder-7 (GAD-7) for anxiety symptoms and the Patient Health Questionnaire-9 (PHQ-9 items) for depression symptoms. Both questionnaires were designed for self-administration and have been validated in the general population [19,20]. The GAD-7 consists of seven items aligned with the most prominent diagnostic features of the DSM-IV diagnostic criteria A, B, and C for generalized anxiety disorder. Cronbach’s α for the entire study sample was 0.9, indicating an excellent internal consistency. Respondents were asked how often, during the last 14 days, they had been bothered by each of the 7 core symptoms of generalized anxiety disorder. Response options were not at all, several days, more than half the days, and nearly every day, scored as 0, 1, 2, and 3, respectively. The total score was divided into 0–4 normal, 5–9 mild anxiety, 10–14 moderate anxiety 15–21 severe anxiety [21].

The PHQ-9 consists of nine items aligned with the DSM-IV Diagnostic Criterion A symptoms for major depressive disorder. Cronbach’s α for the entire sample was 0.87, indicating excellent internal consistency. Respondents were asked how often over the last 14 days they had been bothered by each of the depression symptoms. Response options were not at all, several days, more than half the days, and nearly every day, scored as 0, 1, 2, and 3, respectively. The PHQ-9 total score was divided into 0–4 normal, 5–14 mild major depressive disorder, 15–19 moderate-major depressive disorder, and 20–27 severe major depressive disorder [22].

### 2.4. Data Analysis

Statistical analyses were performed using SPSS Statistic 25.0 (IBM SPSS Statistics, New York, NY, United States). Data from REDCap were directly exported to SPSS. Incomplete surveys were excluded from data analysis to ensure data quality. Descriptive statistics were used to describe the sample sociodemographic characteristics, communication to the personal doctor or other medical advisors and psychological history. Percentages of responses were calculated according to the number of respondents per response concerning the number of total responses of a question. Nonparametric univariate analysis was used to explore the significant associations between sociodemographic characteristics, medical history, and psychological impact of COVID- 19 pandemic. Spearman’s and Pearson’s correlation coefficient, r, was used to evaluate the association between QOL variables, compliance with PM, and the GAD-7 score, as well as the PHQ-9 scores. Statistically significant variables were included in multivariate linear regression models for further investigations. A two-tailed *p* < 0.05 was considered statistically significant, unless otherwise indicated.

## 3. Results

A total of 1652 questionnaires were completed from 3rd to 9th of April. Overall, 919 respondents submitted the questionnaire from day one, 126 respondents submitted it on the second day, 120 on the third day, 128 on the fourth day, 137 on the fifth day, 97 on the sixth day, and 125 respondents submitted the questionnaire on the last recruitment day. Ten unusual cases were detected and removed, and the analysis took place with 1642 participants. A percentage (16.1%) of respondents reported that at the time of their participation were residing abroad. Specifically, 10.8% of the respondents were in Greece, 4.7% were in other European countries, and 0.6% were in North America.

### 3.1. Development of COVID-19 in Cyprus during the Recruitment Period

**Figure 1:**
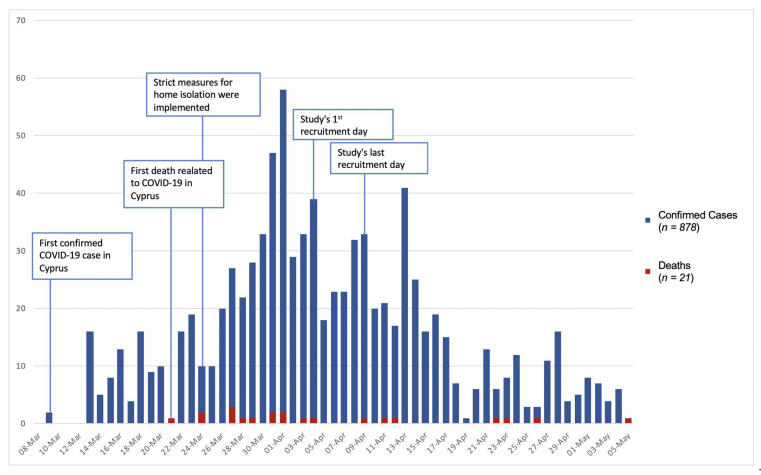
Epidemic trend of Severe Acute Respiratory Syndrome Coronavirus 2 (also known as COVID-19) outbreak in Cyprus from 8 March to 5 May.

Figure 1 shows the development trend of the COVID-19 pandemic in Cyprus during the survey’s recruitment period and the total number of confirmed cases and deaths related to COVID-19 infection per day through May 5th, 2020, the first day after the gradual loosening of the severe lock down measures and the re-start of the Cypriot economy. According to the Cyprus Ministry of Health, until April 3rd, the first day of data collection, there were a total 423 cases with a mean age of 46 years; 6.95% were under age 19, 68.33% were between ages 20–59, and 24.7% were above 60. These patterns were maintained throughout the acute phase of the pandemic.

### 3.2. Anxiety and Depression Levels

The anxiety impact of the COVID-19 pandemic, using the GAD-7, revealed a sample mean score of 6.79 (SD = 4.74). Of all the respondents, 589 (35.9%) were considered to have low GAD score and within the healthy ranges (score: 0–4); a large proportion of the sample 673 (41%) reported mild GAD score (score: 5–9), whereas 230 (14%) of the respondents reported moderate GAD score (score: 10–14), and 150 (9.1%) respondents reported severe GAD score (score: 15–21). 

With regard to the depression symptomatology, PHQ-9 revealed a sample mean score of 6.65 (SD = 5.26). A large number of respondents, 700 (42.6%) scored within the normal ranges (score:0–4 ); 790 (48.1%) reported mild PHQ score (score: 5–14), whereas 102 (6.2%) of the respondents reported moderate PHQ score (score: 15–19) and 50 (3%) respondents reported severe PHQ score (score: 20–27).

### 3.3. Sex and Age Effects

In order to explore the effects of sex and age on anxiety and depression scores, Mann–Whitney test and Kruskal–Wallis tests were used (see Table 1). A Mann–Whitney test indicated that the GAD score was higher for women (Mdn = 6.00) than men (Mdn = 4.00), U = 193370, *p* < 0.001; similarly, the PHQ score was higher for women (Mdn = 6.00) than for men (Mdn = 4.00), U = 229337, *p* < 0.001. There was statistically significant difference in the GAD and PHQ scores reported by different age groups. Kruskal–Wallis H test showed that the youngest group reported the highest anxiety and depression scores (H(4)= 49.671, *p* < 0.001) and H(4) = 148.96, *p* < 0.001, respectively).

### 3.4. Student/Employment Status, Living Status, and Environmental Stressors

Mann–Whitney test and Kruskal–Wallis tests were also used, to explore the effects of other key demographic variables on anxiety and depression (see Table 1). A Mann–Whitney test indicated that the GAD score was greater for students (Mdn = 7.00) than for non-students (Mdn = 5.00), U = 254586, *p* < 0.001. In the same fashion, the PHQ score was greater for students (Mdn = 7.00) than for non-students (Mdn = 5.00), U = 211695, *p* < 0.001.

Those who were employed reported a lower GAD score (Mdn = 6.00) than those who were unemployed (Mdn = 6.00), U = 372155, *p* < 0.001. Similarly, the PHQ score was also significantly lower for those who were employed (Mdn = 5.00) than for those who were unemployed (Mdn = 7.00), U = 409579, *p* < 0.001. 

Of those who were employed (*n* = 956, 58.2% of total participants), the physical/social distancing measures and government lock down regulations resulted in four categories of work force: (1) Those required to maintain their normal work style (*n* = 178, 18.7% of employed), (2) Those who converted to working from home (*n* = 480, 50.1% of employed), (3) Those who did a combination of working from home with occasional trips to the workplace (*n* = 198, of employed), and (4) Those who were self-employed or their companies filed for government assistance to receive the temporary unemployment benefits (*n* = 49, 5.1% of employed). 

A Kruskal–Wallis H test did not reveal significant differences among the different types of work groups on the GAD scale. However, there was a statistically significant difference on the PHQ score among the different work groups (H (4) = 9.788, *p* = 0.04). Participants who were self-employed or their companies filed for government assistance to receive the temporary unemployment benefits reported the highest PHQ scores (mean rank 581.93), followed by those who were employed but did not fall into any of the above groups (mean rank 519.10) and those working from home (mean rank of 462). Frontline workers did not report higher GAD (Mdn = 6) or PHQ scores (Mdn = 5) than non- frontline workers, U = 63346.5, *p* = 0.75, U = 60516.5, *p* = 0.32, respectively.

Living arrangements also affected GAD scores, as those who were living with others (Mdn = 6.00) reported more anxiety symptoms than those who were living alone (Mdn = 5.00), U = 126744, *p* < 0.001. A Kruska–Wallis H test indicated that there was a statistically significant difference in GAD score between the different household size (H (4) = 14.71, *p* = 0.01). Specifically, those who lived with more than 3 people had a statistically higher GAD score (*p* = 0.005) compared to those who lived with only one person. They were not significant differences among the different living arrangements or the different household size on the PHQ scale. 

Closing the borders resulted in a category of individuals who were trapped abroad. Having a first-degree relative trapped abroad was a significant stressor resulting in significantly higher GAD scores (U = 106336, *p* = 0.02), whereas those who were trapped abroad had a statistically significant higher PHQ score (U = 40329, *p* = 0.005) than those not trapped abroad.

### 3.5. Medical History and Psychological Impact

Table 2 shows the associations of medical history and the psychological impact of the COVID-19 pandemic. Overall, 276 respondents (16.8%) reported prior psychiatric history. A Mann–Whitney test indicated that anxiety ratings were higher for those with prior psychiatric history (Mdn = 9.00) than for those without a psychiatric history (Mdn = 5.00), U = 111128.5, *p* < 0.001. Similarly, those with a prior psychiatric history reported a higher depression score (Mdn = 9.00) than those without prior psychiatric history (Mdn = 5.00), U= 118708.5, *p* <0.001. 

Regarding health utilization, about 12.7% of the respondents (*n* = 208) indicated that they communicated with their primary care physician in the two weeks prior to their participation in the study for different medical conditions. Communication with a medical doctor resulted in higher GAD (U = 119090, *p* < 0.00.1) and PHQ scores (U = 135424, *p* = 0.03). However, the specific reasons for the communication were not associated with either the GAD nor the PHQ scores.

### 3.6. Multiple Linear Regression (MLR) Analysis, with Statistically Significant Demographic and Medical History Variables

Results of MLR analysis of factors associated with anxiety and depression are presented in Table 3. Significance variables from the univariate analysis were used for further investigations and to identify predictors to mental health. Being a woman was significantly associated with higher GAD (B = 2.21, 95% CI: 1.71 to 2.7) and PHQ score (B = 1.33, 95% CI: 5.22 to 6.18). Moreover, age was significantly associated with both GAD and PHQ scores. While the youngest group was associated with the highest levels of anxiety, participants ages 30–49 were retained in the equation. The two older age groups (50–59 and 60+) did not predict high levels of anxiety or depression.

Student status was associated with higher GAD (B = 1.47, 95% CI: 0.99 to 1.95) and PHQ scores (B = 3.06, 95% CI: 2.54 to 3.57), with bachelor students being associated with higher anxiety (B = 1.61, 95% CI: 0.26 to 2.78) and depression (B = 3.02, 95% CI: 1.65 to 4.38) scores compared to the PhD students. With regard to the employment status, being employed was associated with lower GAD scores (B = −1.15, 95% CI: −1.61 to 0.69) and PHQ score (B = −2.44, 95% CI: −2.94 to 1.94). 

Living with others was associated with a greater number of GAD symptoms (B = 0.83, 95% CI: 0.14 to 1.53) compared to living alone, whereas house-hold size was not predictive of neither with GAD nor PHQ scores. Other stressors, such as having a first-degree relative trapped abroad (B = 1.13, 95% CI: 0.26 to 1.99) or been trapped abroad (B = 2.63, 95% CI: 0.98 to 4.28), were found to be significant predictors of GAD and PHQ, respectively. 

Prior psychiatric history was found to be significant predictor of both GAD (B = 3.65, 95% CI: 3.06 to 4.23) and PHQ (B = 3.69, 95% CI: 3.03 to 4.35) scores. Health utilization in the form of communicating with a medical doctor was also a significant predictor of both GAD (B = 1.76, 95% CI: 1.07 to 2.45) and PHQ (B = 0.87, 95% CI: 0.11 to 1.64) scores.

### 3.7. Quality of Life (QOL) and Psychological Impact 

Correlation analyses was conducted between QOL, anxiety and depression scores. The results suggest that each question regarding QOL was statistically correlated with anxiety and depression scores (*p* < 0.001). A composite score of QOL was calculated and used as an independent variable, since all the QOL-related questions were intercorrelated and moderately-highly correlated to the QOL total score (*r* ≥ 0.49, *p* < 0.001)

Multiple linear regression (MLR) was carried out to predict GAD score based on the QOL composite score, sex, and age. The analysis resulted in a statistically significant model, (F (3.1638) = 238.64, *p* < 0.001) with R2 of 0.304. All three variables were significant predictors of GAD and PHQ and were retained in the model, *p* < 0.001. Specifically, the GAD score increased by 0.77 points for each point of QOL increase (indicating lower levels of QOL), by 1.67 points for being a woman and by 0.54 points for being in the youngest age group of 18–29. 

Another MLR was carried out to predict the PHQ score based on the same independent variables as the above. The regression model was found to be significant (F(3, 1638) = 198.29, p < 0.001) with R2 of 0.266. Two out of the three variables were retained in the model, *p* < 0.001; sex was not a statistically significant predictor. Participants’ PHQ score increased by 0.75 points for each point of QOL increase (indicating lower levels of QOL) and by 1.14 points for belonging in the youngest age group of 18–29.

### 3.8. Compliance with Precautionary Measure (PM) and Psychological Impact

A principal component analysis (PCA) was run on the 12 questions that measured compliance with PM. The suitability of PCA was assessed prior to analysis. Inspection of the correlation matrix showed that all variables had at least one correlation coefficient greater than 0.3. The overall Kaiser-Meyer-Olkin (KMO) measure was 0.85. Bartlett’s test of sphericity was statistically significant (p < 0.001), indicating that the data was likely factorizable. PCA revealed two components that had eigenvalues greater than one, explaining 29.38% and 11.84% of the total variance, respectively. Visual inspection of the scree plot indicated that the two components can be retained. A Varimax orthogonal rotation was employed to aid interpretability. As it can be seen in Table 4, the 1st component was labeled “Personal hygiene and Indoors-related precautionary measures” due to the high loadings with items, such as, “I wash my hands regularly with water and soap for at least 20 s”, or “I use antiseptic whenever it is not possible to wash my hands with soap and water”. The 2nd component was labeled “Outdoors-related precautionary measures” due to the high loadings by items, such as: “I avoid handshakes and other behaviors (e.g., hugs) that require coming in physical contact with other people outside the house”, “I avoid consuming takeaway food”, etc. Component loadings and communalities of the rotated solution are presented in Table 4.

Correlational analyses examined the relationship between compliance with PM and the anxiety and depression scale scores. Overall, there was an inverse weak but significant relationship between the total compliance with PM score and the score on the PHQ scale (*r* = −0.11, *p* < 0.001). PM individual factors were also correlated with mental health indices. Factor 1, “Personal hygiene and Indoors-related precautionary measures” was positively correlated with the GAD scale score (*r* = 0.06, *p* = 0.02) and negatively correlated with the PHQ scale score (*r* = −0.09, *p* < 0.001), where’s Factor2, the “Outdoors-related precautionary measures” factor was negatively correlated with the PHQ scale score (*r* = −0.09, *p* < 0.001). 

Three different MLR were carried out to determine if sex and age predicted compliance with the PM total score and the two factors, “Personal hygiene and Indoors-related precautionary measures” and “Outdoors-related precautionary measures”. Sex and age significantly predicted compliance with PM total score (F (2.1632) = 110.53, *p* < 0.001) with R2 of 0.12. Similarly, sex and age were significant predictors for compliance with the “Personal hygiene and Indoors-related precautionary measures” factor, (F (2.1632) = 104.02, *p* < 0.001) and with the “Outdoors-related precautionary measures” factor, (F (2.1632) = 42.66, *p* < 0.001) with R2 = 0.11 and R2 = 0.05. Specifically, women and participants 30 years and above were more engaged with PM. 

## 4. Discussion

The present study provides important information on the impact of COVID pandemic in the general population, about two weeks into the implementation of restrictive government measures from a large Mediterranean cohort. Cyprus is a small geographical area coupled with unique cultural, genetic, and societal characteristics stemming from a rich 10,000 years history, rendering it conducive to health care research. The outcomes of the present study inform the literature with important evidence on the impact of the pandemic during the acute phase and provide direction to policy makers on the potential long-term needs that will arise after as the severe lock down measures begin to lift.

First, we found significant elevation of COVID-19 related anxiety and depression symptoms (64.1% and 57.3%, respectively). In particular, 23.1% of the respondents reported moderate-severe anxiety symptoms, and 9.2% reported moderate-severe depression symptoms. Second, the presence of psychological morbidity was associated with being a woman, under 50, a university student, and/or unemployed. Those living with others, being trapped abroad or having a first order relative trapped abroad, also had increased risk. Third, psychological morbidity was further associated with other medical conditions, such as prior psychiatric history and health utilization during the pandemic. Finally, QOL was significantly hampered by the pandemic, and reduced QOL was a significant predictor of psychological morbidity.

The implementation of PM has been a critical aspect for the control of COVID-19 pandemic. Our findings indicate that greater levels of compliance with PM were related to lower depression scores. At the same time, compliance with certain aspects of PM was also related with increased levels of anxiety. Furthermore, women and participants above 30 years old were more likely to engage in any kind of PM than men or younger participants under 30. In the next sections, we provide more in-depth discussion of the study findings and implications for public health.

### 4.1. Prevalence of Anxiety and Depression and the Role of Sex and Age

The study findings expand our limited knowledge about the psychological effects of the COVID-19 pandemic [16,17] and raise significant concerns regarding potential lingering effects of these immediate responses. The rate of anxiety and depression levels in the Cypriot cohort was significantly elevated due to the pandemic, with 23.1% reporting moderate-severe anxiety, and 9.2% reporting moderate-severe depression scores. This is in contrast to the 1% and 3.9%, respectively, which is the prevalence reported in the European Study of the Epidemiology of Mental Disorders during normal conditions [23]. Wang et al. (2020) in their study taking place during the initial state of the pandemic in China reported even higher rates than Cyprus, with 28.8% of the sample reporting moderate-severe anxiety and 6.5% reporting moderate-severe depression symptoms. One interpretation might be that the Chinese population was primed due to the SARS 2003 and subsequent H1N1 pandemic in 2009. The Cypriot population had not experienced a health pandemic in decades; the last traumatic event that caused death and affected the entire country in a similar diffuse manner were the two Turkish invasions in 1974. Additional studies from other European countries will provide more information on potential differences in the prevalence of symptoms among countries and across continents.

As expected, there were sex differences in the symptom distribution, with women reporting significantly higher levels of anxiety and depression than men. This finding can be supported by many epidemiological studies, reporting that women are at a higher risk for developing anxiety and depression [23,24,25]. Therefore, sex patterns in the distribution of symptoms were maintained during the pandemic.

Age was another important sociodemographic variable since the youngest group ages 18 to 29 demonstrated significantly more mental symptoms. This finding corresponds to previous related works that support a negative correlation between age and anxiety and/or depression symptoms [26]. A possible explanation is that age provides opportunities to build resilience due to exposure to multiple and different stressors across time, resulting in better emotional management and lower anxiety and depression symptoms [27]. 

A large percentage of younger study participants were also university students. Student status was also associated with higher anxiety and depression rates as compared to non-student status. Additionally, undergraduate students reported significantly higher levels of anxiety and depression than graduate or doctoral students. The current findings expand the results from the published study from China on COVID-19 which included only undergraduate students. In that study, university students also reported high rates of anxiety and depression [16,17]. While part of the elevated symptoms could be accounted by age, as most university students are typically under 30 years of age, the increased negative impact on mental health could be attributed to sudden lifestyle changes and disruption of regular social activities. Additional stressors include impromptu replacement of face-to-face classes to e-learning and, in several cases, changes in the living arrangements. According to prior research, e-learning results in negative reactions by university students who, as a group, prefer face-to-face learning [28,29]. Overall, the new reality created uncertainty on how the pandemic and distancing measures would impact final examinations and class assignments, as well as how the pandemic would affect the future of their studies. Therefore, uncertainty could have contributed to the significantly elevated scores, especially within the undergraduate participants.

### 4.2. Social Determinants of Anxiety and Depression

The COVID-19 pandemic and measures to control it had a great impact on OQL for more than 65% of the study sample. Key aspects included worry about finances, worry about personal health, satisfaction with current state of QOL, and current levels of health. Equally important is the fact that only 28% of the sample indicated satisfaction with QOL. Higher levels of dissatisfaction were positively associated with a greater number of reported symptoms of anxiety and depression. Our results are in line with previous studies, showing the interplay between QOL and psychological symptoms in the general population, independent of age and education [30,31,32]. Furthermore, our results provide important information on social determinants that impact QOL and the need to develop mechanisms to support the public.

An important determinant of mental health was the financial instability and uncertainty created by the lock down measures and the pandemic. Over 48% of study participants reported significant concerns about the impact of the pandemic on their personal/family finances. Those participants whose employers required them to work from home due to social-physical distancing measures and received their regular salaries experienced the least amount of symptoms, as compared to those who were forced into unemployment and welfare benefits. Unemployment and welfare benefits were announced by the Cypriot Government a few days prior to the onset of data collection. The benefits were granted to support the majority of the private sector in Cyprus, the self-employed, and small- to medium-sized enterprises. Despite these announcements and plans for support, this group of participants was impacted the most. While the government would support a percentage of their salary ranging from 50–70% depending on the eligible benefit scheme, the pandemic and lock down measures created significant uncertainly on critical aspects of financial viability, such as the timing of the welfare payment and their ability to recover the remaining percentage of their salary, as well as duration of the government benefits. Previous research suggests that involuntary unemployment result in higher levels of anxiety and depression [33,34]. Other important factors, such as worry about their future job stability and income potential, could contribute to the higher levels of anxiety and depression in that particular group. Finally, the widespread concern on finances reported by 48% of the respondents may also be linked to the 2011–2015 financial crisis characterized by the infamous haircut of bank accounts in 2013, business closings, job losses, brain drain, and widespread financial repercussions. Salary reductions continue to be in effect to date for all employees in the public and semi-public sectors, as well as some sectors of the private industry [35,36]. 

An unexpected finding of the present study was that those participants who were living with others had increased anxiety compared to those who lived alone. This finding is in contrast with previous studies, since living alone increasing the risk for development of common mental disorders, such as anxiety and depression [37,38]. One possible explanation is that individuals who lived with others during the pandemic may have experienced higher levels of stress due to concerns about transmitting the virus to others and/or contracting the virus from others as opposed to people who lived alone. This is especially relevant if they lived with individuals who belonged in vulnerable groups for the COVID-19 or with individuals who did not adhere closely to the PM. Further studies are needed to investigate these hypotheses. 

### 4.3. Compliance with COVID-19 Precautionary Measures (PM) and Implications for Public Health

The present study examined compliance with twelve key PM to safeguard personal and public health and prevent the spread of COVID-19. Overall, the present study demonstrates that implementation of the PM was predictive of lower scores on depression symptoms. Similarly, the Wang et al. (2020) study from the recent pandemic in China also reported that the implementation of PM by study participants had a protective psychological effect. We assert that implementing PM as suggested by the WHO [1] and other important authorities, such as the European Centers for Disease Prevention and Control and the Cyprus Government, may have provided safety and control to study participants at the time of the pandemic.

On the other hand, our study indicated that very high adherence to the personal hygiene factor (i.e., hand washing, use of disinfectant, wiping down knobs and surfaces, etc.) was predictive of higher levels of anxiety. During the SARS pandemic, Leung et al. (2003) also reported that participants with moderate levels of anxiety were most likely to take comprehensive PM against the infection. The ongoing bombardment by all media outlets on the disastrous effects of the COVID-19 pandemic and the need to adhere to PM may have contributed to the association between anxiety levels and adherence to personal hygiene. Personal hygiene was a primary personal safety mechanism to gain control over the infection.

Adherence to the PM by the general public was a critical aspect in order to control the spread of the disease during the climax of the pandemic. Adherence to the PM continues to be of outmost public health concern in order to keep the spread of the disease at bay and prevent a second wave of the pandemic in the fall. Governments ask their citizens to honor these measures as a second lock-down would be detrimental to the national and international economy. Our findings indicate women and adults over the age of 30 were more likely to implement the measures. The present findings provide important information to public authorities in order to develop programs and campaigns targeting the groups least likely to adhere to PMs.

### 4.4. Study Limitations, Implications, and Future Research

The present study was a large internet-based cohort study. Results cannot be generalized to individuals over 60 as the majority of the sample was ages 18–60. We elected to conduct an anonymous survey in order to ensure maximum disclosure by study participants. This approach prevented us from conducting follow up studies with the same sample or to track individuals at a high risk for anxiety and depression.

The present study has important implications for public health. The release of the survey was carefully timed to capture the effects during the climax of the pandemic and about two weeks after the implementation of the severe restrictions in movement. While the measures were deemed necessary and were successful in managing the public health threat of COVID-19, the measures had widespread effects on mental health and well-being as demonstrated by the study findings. The results can guide policy makers, such as ministries of health and labor, to create programs in the wider community and manage the psychosocial problems arising from the pandemic and prevent secondary persistent effects. 

In the current cohort, about 16% reported of having a prior history of mental health illness. Our analysis indicates that participants with prior psychiatric history are more vulnerable to present severe anxiety and/or depression symptoms compared to the rest of the population. This group in particular, should be of great concern to public health and mental health professionals in order to provide targeted support and prevent more severe mental health relapses.

Moreover, our study indicates the need for development of specific programs in order ensure proper adherence to PM in the general public, especially for the groups with low current uptake of precautions, such as individuals under 30 years of age and males. This is of outmost relevance, not only as citizens resume more normal activities, but also in the anticipation of a second wave of the disease, and in developing crisis intervention plans for future disasters.

The cost of mental disorders in 2010 for Europe was estimated at € 523.3 billion [39], whereas worldwide costs exceed US $ 16.1 trillion [40]. The present findings suggest that the COVID-19 pandemic may contribute to the development of additional mental health burden. The data provided can guide employers on anticipating potential difficulties faced by their associates and employees during the second phase of the pandemic and during the critical phase of restarting the economy. Through the development of psychosocial support mechanisms to manage anxiety, stress, and depression, we could prevent secondary health effects of the pandemic, loss of productivity, reduction in QOL, and overreliance on healthcare and social welfare systems.

## 5. Conclusions

Our findings confirm that the pandemic and the implementation of the restrictive measurements had great impact on the psychological state and the QOL of the general population. Women, people of younger age, student status, unemployment status, prior psychiatric history, and those reporting greater negative impact on their QOL were at higher risk for increased anxiety and depression symptoms. Moreover, high engagement with PM might act as a protective factor for depression, but specific PM related to personal hygiene might lead to increased anxiety symptoms. Younger adults and males reported lower adherence to PM as compared to females and adults over 30. Our findings highlight the need for psychological interventions to help those with lingering mental health difficulties as they reintegrate into their pre-COVID-19 lifestyle. The findings also point to the need for targeted programs to ensure compliance with PM and prevent disease transmission and future outbreaks of COVID-19. 

## Figures and Tables

**Table 1:** Relationship between Sociodemographic Characteristic of the sample with anxiety and depression scores.

Characteristics	*n* (1642)	Anxiety				Depression			
	*n* (%)	Mean	SD	Statistics	*p*-Value	Mean	SD	Statistics	*p*-Value
Sex									
Women	1176 (71.60)	7.42	4.80	193370 ^a^	<0.001	7.03	5.34	229337 ^a^	<0.001
Men	466 (28.40)	5.21	4.18			5.70	4.94		
Age group									
18 to 29	696 (42.30)	7.61	4.93	49.671 ^b^	<0.001	8.47	5.79	148.966 ^b^	<0.001
30 to 39	365 (22.20)	6.70	4.52			5.64	4.08		
40 to 49	311 (18.90)	6.01	5.45			5.45	4.89		
50 to 59	208 (12.70)	5.88	4.47			4.24	3.57		
60 plus	63 (3.80)	4.83	4.22			3.90	3.57		
Studying status									
Yes	578 (22.00)	7.74	4.98	254586 ^a^	<0.001	8.63	5.94	211695 ^a^	<0.001
No	1064 (64.80)	6.28	4.52			5.57	4.51		
Employment status									
Yes	956 (58.20)	6.31	4.52	372155 ^a^	<0.001	5.63	4.58	409579 ^a^	<0.001
No	686 (41.80)	7.46	4.95			8.08	5.80		
Living circumstances									
Living with others	1437 (87.50)	6.89	4.7	126744 ^a^	0.001	6.61	5.21	150338 ^a^	0.63
Alone	205 (12.50)	6.06	4.92			6.93	5.67		
Number of people living with									
Plus 1	276 (26.80)	6.22	4.75	14.71 ^b^	0.005	6.17	5.14	10.75 ^b^	0.03
Plus 2	355 (21.60)	6.64	4.57			6.07	4.83		
Plus 3	422 (25.70)	7.35	4.71			6.88	5.18		
Plus 4	258 (15.70)	6.98	4.8			7.25	5.90		
Plus 5 or more	134 (9.30)	6.87	4.70			6.61	5.20		
Other Sociodemographic status									
Frontline worker									
Yes	83 (5.10)	6.60	4.84	63346.5 ^a^	0.75	6.69	5.29	60516.5 ^a^	0.32
No	1559 (94.90)	6.80	4.73			5.95	4.64		
First degree relative trapped abroad									
Yes	124 (7.60)	7.83	4.99	106336 ^a^	0.02	7.37	5.71	100872 ^a^	0.18
No	1518 (92.40)	6.70	4.71			6.59	5.22		
Trapped abroad status									
Yes	40 (2.40)	7.50	5.03	34648.5 ^a^	0.38	9.22	6.21	40329 ^a^	0.005
No	1602 (97.60)	6.77	4.73			6.59	5.22		
Students education level									
Bachelor	362 (22.0)	8.22	5.03	10.713 ^b^	0.005	9.44	5.87	23.321 ^b^	<0.001
Master	128 (7.80)	7.16	5.01			7.89	6.44		
Ph.D	88 (5.40)	6.61	4.48			6.42	4.67		
Workplace protection measures									
“I’m working from home“	480 (29.20)	6.11	4.34	4.044 ^b^	0.4	5.36	4.42	9.788 ^b^	0.04
“I sometimes work from home and sometimes at my workplace”	198 (12.10)	6.22	4.26			5.51	4.29		
“I’m still working at my workplace”	178 (10.80)	6.57	5.11			5.91	5.01		
“I’m out of work and will be paid 60% of my salary”	49 (3.00)	6.77	4.64			7.38	5.27		
Other	53 (3.20)	7.28	4.88			5.64	4.59		

^a^ Mann–Whitney test, ^b^ Kruskal–Wallis test.

**Table 2:** Relationship between Medical History variables with anxiety and depression scores.

Medical History	*n* (1642)	Anxiety				Depression			
	*n* (%)	Mean	SD	Statistics	*p*-Value	Mean	SD	Statistics	*p*-Value
Prior psychiatric history									
Yes	276 (16.80)	9.82	5.29	111128.5 ^a^	<0.001	9.72	6.09	118708.5 ^a^	<0.001
No	1366 (83.20)	6.18	4.37			6.03	4.86		
Communication with medical doctor									
Yes	208 (12.70)	8.33	5.22	119090 ^a^	<0.001	7.41	5.55	135424 ^a^	0.03
No	1434 (87.30)	6.57	4.62			6.54	5.21		
Reason for communication									
Flu Symptoms, COVID-19	50 (3.00)	8.08	4.53	2.927 ^b^	0.71	7.50	5.25	3.067 ^b^	0.69
Gastrointestinal	6 (0.40)	7.33	3.93			6.33	3.38		
Musculoskeletal	7 (0.40)	6.14	4.22			4.57	5.32		
Cardiovascular	4 (0.20)	8.25	8.77			6.75	1.70		
Pulmonar	17 (1.00)	8.82	3.57			6.88	3.55		
Other	126 (7.70)	8.49	5.64			7.65	6.04		

^a^ Mann-–Whitney test, ^b^ Kruskal–Wallis test.

**Table 3:** Association between statistically significant variables with anxiety and depression scores.

Characteristics	N(1641)	Anxiety			Depression		
	*n* (%)	R^2^	AR^2^	B (95% CI)	R^2^	AR^2^	B (95% CI)
Sex		0.04	0.04		0.01	0.01	
Woman	1176 (71.60)			2.21 *** (1.71 to 2.7)			1.33 *** (5.22 to 6.18)
Man	466 (28.40)						
Age group		0.03	0.03		0.09	0.09	
18 to 29	696 (42.30)			2.79 *** (1.58 to 3.99)			4.57 *** (3.28 to 5.87)
30 to 39	365 (22.20)			1.87 ** (0.61 to 3.13)			1.73 * (0.39 to 3.09)
40 to 49	311 (18.90)			1.25 * (−0.02 to 2.52)			1.55 * (0.19 to 2.91)
50 to 59	208 (12.70)			1.06 (−0.26 to 2.38)			1.09 (−0.33 to 2.50)
60 plus	63 (3.80)						
Student status		0.02	0.02		0.08	0.08	
Yes	578 (22.0)			1.47 *** (0.99 to 1.95)			3.06 *** (2.54 to 3.57)
No	1064 (64.8)			-			-
Employed status		0.01	0.01		0.05	0.05	
Yes	956 (58.20)			−1.15 *** (−1.61 to −0.69)			−2.44 *** (−2.94 to −1.94)
No	686 (41.8)						
Household size		0.003	0.003				
Living with others	1437 (87.50)			0.83 * (0.14 to 1.53)			
Alone	205 (12.05)						
Number of people living with							
Plus 1	276 (19.10)	0.01	0.01	−0.96 (−1.92 to 0.01)			
Plus 2	355 (24.60)			−0.54 (−1.47 to 0.39)			
Plus 3	422 (29.20)			0.17 (−0.74 to 1.08)			
Plus 4	258 (17.90)			−0.19 (−1.19 to 0.78)			
Plus 5 or more	134 (9.30)			-			
Other Sociodemographic status							
First degree relative trapped abroad							
Yes	124 (7.60)	0.004	0.003	1.13 * (0.26 to 1.99)			
No	1518 (92.40)						
Trapped abroad status							
Yes	40 (2.40)				0.01	0.01	2.63 ** (0.98 to 4.28)
No	1602 (97.60)						
Students education level							
Bachelor	362 (22.00)	0.02	0.01	1.61 ** (0.26 to 2.78)	0.04	0.03	3.02 *** (1.65 to 4.38)
Master	128 (7.80)			0.55 (−0.79 to 1.89)			1.48 (−0.11 to 3.07)
PhD	88 (5.40)						
Workplace protection measures							
“I’m working from home“	480 (29.20)				0.01	0.01	−0.96 (2.26 to 0.34)
“I sometimes work from home and sometimes at my workplace”	198 (12.10)						−0.81 (−2.21 to 0.57)
“I’m still working at my workplace	178 (10.80)						−0.41 (−1.81 to 0.99)
“I’m out of work and will be paid 60% of my salary”	49 (3.00)						1.07 (−0.71 to 2.85)
Other	53 (3.20)						
Medical History							
Prior psychiatric history							
Yes	276 (16.80)	0.08	0.08	3.65 *** (3.06 to 4.23)	0.07	0.07	3.69 *** (3.03 to 4.35)
No	1366 (83.20)						
Communication with medical doctor (within the past 2 weeks)							
Yes	208 (12.70)	0.02	0.02	1.76 *** (1.07 to 2.45)	0.003	0.002	0.87 * (0.11 to 1.64)
No	1434 (87.30)						

* *p* < 0.05; ** *p* < 0.01; *** *p* < 0.001.

**Table 4:** Rotated Structure Matrix for PCA with Varimax rotation of a two Component Questionnaire.

Items	Rotated Component Coefficient	
	Personal Hygiene and Indoors-Related Precautionary Measures	Outdoors-Related Precautionary Measures
I disinfect surfaces I use regularly (e.g., doorknobs, switches, etc.)	**0.78**	0.10
I wash my hands regularly with water and soap for at least 20 s	**0.70**	0.01
I use antiseptic whenever it is not possible to wash my hands with soap and water	**0.68**	−0.01
I avoid hand contact with my mouth, nose or eyes	**0.65**	0.16
When I am indoors, I make sure there is adequate natural ventilation	**0.60**	0.22
I cover my mouth and nose with a tissue or my sleeve (not my hand) when I cough or sneeze	**0.51**	0.18
On a daily basis, I spend time following different media outlets (e.g., internet, TV, etc.) to learn about COVID-19 updates	**0.37**	0.11
I am on self-quarantine at home and I avoid places of social gatherings	0.02	**0.73**
I avoid handshakes and other behaviors (e.g., hugs) that require coming in physical contact with other people outside the house	0.23	**0.69**
I avoid consuming takeaway food	0.20	**0.62**
Outside the house, I maintain at least one-meter distance from other people	0.37	**0.50**
I leave the house only when absolutely necessary (e.g., work, supermarket, etc.)	−0.02	**0.47**

Bold text indicates items loading on each component.
